# A Systematic Review on PD-1 Blockade and PD-1 Gene-Editing of CAR-T Cells for Glioma Therapy: From Deciphering to Personalized Medicine

**DOI:** 10.3389/fimmu.2021.788211

**Published:** 2022-01-19

**Authors:** Mahdi Abdoli Shadbad, Nima Hemmat, Vahid Khaze Shahgoli, Afshin Derakhshani, Farzad Baradaran, Oronzo Brunetti, Rossella Fasano, Renato Bernardini, Nicola Silvestris, Behzad Baradaran

**Affiliations:** ^1^ Student Research Committee, Tabriz University of Medical Sciences, Tabriz, Iran; ^2^ Immunology Research Center, Tabriz University of Medical Sciences, Tabriz, Iran; ^3^ Research Center for Evidence-Based Medicine, Tabriz University of Medical Sciences, Tabriz, Iran; ^4^ Cancer and Inflammation Research, Department of Molecular Medicine, University of Southern Denmark, Odense, Denmark; ^5^ Laboratory of Experimental Pharmacology, IRCCS Istituto Tumori Giovanni Paolo II, Bari, Italy; ^6^ Department of Computer (Computer engineering–Artificial Intelligence), Shabestar Branch, Islamic Azad University, Shabestar, Iran; ^7^ Medical Oncology Unit, IRCCS Istituto Tumori “Giovanni Paolo II” of Bari, Bari, Italy; ^8^ Department of Biomedical and Biotechnological Sciences, University of Catania, Catania, Italy; ^9^ Department of Biomedical Sciences and Human Oncology (DIMO), University of Bari, Bari, Italy; ^10^ Department of Immunology, Tabriz University of Medical Sciences, Tabriz, Iran; ^11^ Pharmaceutical Analysis Research Center, Tabriz University of Medical Sciences, Tabriz, Iran

**Keywords:** glioma, CAR-T cells, engineered cell therapy, inhibitory immune checkpoint, single-cell sequencing, tumor microenvironment, neoantigen, personalized medicine

## Abstract

**Background:**

Programmed cell death protein 1 (PD-1) can attenuate chimeric antigen receptor-T (CAR-T) cell-mediated anti-tumoral immune responses. In this regard, co-administration of anti-PD-1 with CAR-T cells and PD-1 gene-editing of CAR-T cells have been suggested to disrupt this inhibitory axis. Herein, we aim to investigate the advantages and disadvantages of these two approaches and propose a novel strategy to ameliorate the prognosis of glioma patients.

**Methods:**

Scopus, Embase, and Web of Science were systematically searched to obtain relevant peer-reviewed studies published before March 7, 2021. Then, the current study was conducted based on the preferred reporting items for systematic reviews and meta-analyses (PRISMA) statements. The random-effect model was applied to evaluate the effect size of administrated agents on the survival of animal models bearing gliomas using RevMan version 5.4. The Cochran Q test and I^2^ were performed to assess the possible between-study heterogeneity. Egger’s and Begg and Mazumdar’s tests were performed to objectively assess potential asymmetry and publication bias using CMA version 2.

**Results:**

Anti-PD-1 can substantially increase the survival of animal models on second-generation CAR-T cells. Also, PD-1 knockdown can remarkably prolong the survival of animal models on third-generation CAR-T cells. Regardless of the CAR-T generations, PD-1 gene-edited CAR-T cells can considerably enhance the survival of animal-bearing gliomas compared to the conventional CAR-T cells.

**Conclusions:**

The single-cell sequencing of tumoral cells and cells residing in the tumor microenvironment can provide valuable insights into the patient-derived neoantigens and the expression profile of inhibitory immune checkpoint molecules in tumor bulk. Thus, single-cell sequencing-guided fourth-generation CAR-T cells can cover patient-derived neoantigens expressed in various subpopulations of tumoral cells and inhibit related inhibitory immune checkpoint molecules. The proposed approach can improve anti-tumoral immune responses, decrease the risk of immune-related adverse events, reduce the risk of glioma relapse, and address the vast inter-and intra-heterogeneity of gliomas.

## 1 Introduction

High-grade gliomas are among the most common primary brain tumors; however, the current therapies have not led to meaningful outcomes for affected patients. Tumor invasion, heterogeneity, and immune escape are considered the daunting challenges for treating these highly aggressive tumors. Therefore, there is a pressing need to develop a safe and effective therapy for patients with high-grade gliomas (1).

Immunotherapy has offered a new treatment approach for some cancers; however, the overall low response rates of immunotherapy for some solid cancers have limited their widespread clinical translations. As “living drugs”, CAR-T cells are engineered cells that can specifically target defined antigens expressed by tumor cells. The ectodomain of CAR-T cells consists of a single-chain variable fragment (scFv) that recognizes tumor-associated antigens and leads to the activation of its endodomain, CD3ζ. The endodomain of the first CAR-T cell generations does not contain other co-stimulatory factors besides CD3ζ. However, the second generation of CAR-T cells has other endodomain co-stimulatory components, i.e., CD28, CD137, or CD134. While the third-generation CAR-T cells were developed by adding two co-stimulatory factors to the CD3ζ, the fourth-generation ones were genetically edited to express desired factors following stimulation (2). Despite the food and drug administration (FDA) approval for CD19-targeting CAR-T cells in patients with B-cell malignancies, CAR-T cell therapy for other solid cancers has not been as promising (3).

Although the addition of co-stimulatory factors to the first generation of CAR-T cells has shown promising results in stimulating anti-tumoral immune responses, the immunosuppressive tumor microenvironment is now recognized as a critical culprit for the low response rates of CAR-T cells in solid cancers (4, 5). Indeed, the expression of inhibitory immune checkpoints, e.g., PD-1, on the CAR-T cells has been associated with a remarkable decrease in their ability to target tumoral cells (6). A recent clinical trial has shown that anti-EGFRVIII-CAR-T cell infusion can substantially promote immunosuppressive tumor microenvironment *via* upregulating inhibitory immune checkpoint molecules (7). In this regard, two approaches have been proposed to suppress PD-1 expression, i.e., PD-1-targeting monoclonal antibodies co-administration with CAR-T and PD-1 gene editing of CAR-T cells (8–11).

Here, we review the current evidence on the efficacy and safety of the combined therapy of PD-1-targeting monoclonal antibodies co-administration with CAR-T cells and PD-1 gene editing of CAR-T cells. Besides, we review the current trend in CAR-T cells therapy of high-grade gliomas in clinical trials and propose a novel strategy for immunotherapy of high-grade gliomas based on preclinical and clinical findings. Our proposed approach is based on the combination of fourth-generation CAR-T cell and single-cell sequencing technologies, which can address the shortcomings in terms of the safety and efficacy of CAR-T cells/immune checkpoint inhibitors for treating patients with high-grade gliomas.

## 2 Material and Methods

This study was conducted under the PRISMA statements (12).

### 2.1 The Search Strategy

Without imposing any restriction on the publication language and publication time, the Web of Science, Scopus, and Embase were systematically searched to obtain records published before March 7, 2021, with the following keywords: (“glioma” OR “glioblastoma” OR “glioblastoma multiforme”) and (“programmed cell death 1” OR “PD-1” OR “PDCD1” OR “hSLE1” OR “CD279” OR “PD1” OR “SLEB2” OR “hPD-l” OR “programmed death 1 receptor” OR “hPD-1”) and (“chimeric antigen receptor T-cell immunotherapy” OR “CAR-T” OR “CART” OR “CAR T” OR “CAR T cell” OR “chimeric antigen receptor T cell” OR “adoptive immunotherapy” OR “chimeric antigen receptor immunotherapy” OR “chimeric antigen receptor T”). We also used the Emtree terms to increase the sensitivity of our systematic search.

### 2.2 Eligibility Criteria

Studies with the following eligibility criteria were included in our study: (1) investigations that studied the effect of CAR-T cells on glioma, and (2) investigations with the objective of evaluating PD-1 suppression on the efficacy of CAR-T cells. Based on the following criteria, studies were excluded from the current systematic review: (1) studies that did not meet the abovementioned inclusion criteria, (2) review papers, (3) meeting abstracts, (4) perspectives, (5) book chapters, (6) editorial articles, (7) commentaries, (8) opinion articles, and (9) duplicated papers.

### 2.3 Study Selection

Following the systematic search, the retrieved records were reviewed in two phases. In the first phase, the titles and abstracts of obtained papers were screened. In the second phase, the full text of the remaining papers and their supplementary data were reviewed for consideration to be included in the current study.

### 2.4 Data Extraction

The following data were extracted from the included studies: (1) the first author, (2) the year of publication, (3) the method of PD-1 suppressing, (4) their main findings, (5) the target of CAR-T cells, (6) the glioma cell line, (7) the schedule of anti-PD-1 administration in animal models, and (8) animal models. For the survival analysis, we extracted the hazard ratio (HR) and the 95% confidence interval (CI) for further analysis.

### 2.5 Evaluating the Quality of Included Studies

To enhance transparency and facilitate the translation of our results into the clinic, we used three quality assessment tools for evaluating the quality of clinical, *in vivo*, and *in vitro* studies. For our included clinical study, we used the “NIH quality assessment tool” (https://www.nhlbi.nih.gov/health-topics/study-quality-assessment-tools). For our included *in vivo* studies, we utilized the “SYRCLE’s RoB” tool, adapted from the Cochrane RoB tool (13). For our included *in vitro* studies, we adapted the previously used quality assessment tool (14, 15).

### 2.6 Statistical Analysis

All meta-analyses were conducted using RevMan version 5.4. Because there might be unpublished investigations, the random-effect model was applied for the current meta-analysis. To objectively evaluate the effect of administrated agents on the survival of mice bearing glioma, the common effect sizes were calculated based on the obtained HRs from included studies. The standard chi-squared test and I^2^ statistics were applied to evaluate potential heterogeneity between the included studies. The values over 75% for I^2^ were considered considerable heterogeneity (16, 17). To assess the potential publication bias, funnel plots were provided using CMA version 2. Besides, Begg and Mazumdar’s test was conducted to assess the potential publication bias objectively. Also, Egger’s test was performed to evaluate potential publication bias statistically.

## 3 Results

### 3.1 Selected Studies

**Figure 1:**
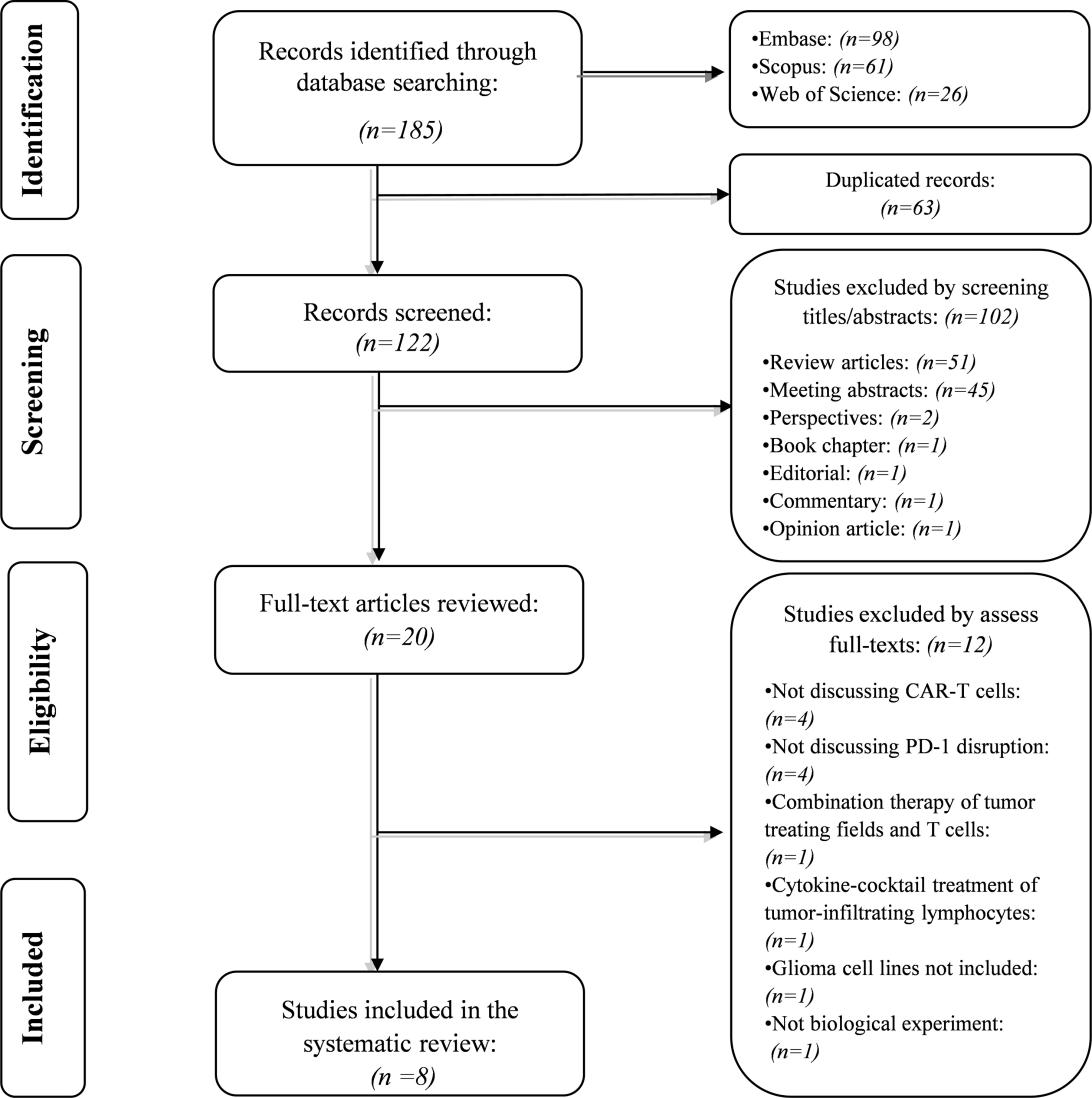
The flow chart of the current study.

Our systematic search retrieved 185 records: Embase (*n*= 98), Scopus (*n*= 61), and Web of Science (*n*= 26). After removing the duplicated studies, 122 studies were screened based on their title and abstracts. In the first phase, 102 records were excluded because they did not meet the abovementioned criteria. In the second phase, the full text of 20 studies and their supplementary data were reviewed for consideration to be included in the systematic review. After excluding twelve studies, we included eight studies in the current systematic review. The flowchart of literature identification is shown in **Figure 1**.

### 3.2 The Characteristics of Included Studies

The eight included studies were published in English between 2018 and 2021. One of the studies was from a phase I clinical trial, and the others were preclinical investigations. Four studies used monoclonal antibodies to block PD-1, and the other four studies inhibited PD-1 gene expression in CAR-T cells. The targets of CAR-T cells were epidermal growth factor receptor variant III)EGFRvIII(, interleukin 13 receptor alpha 2)IL13Rα2), human epidermal growth factor receptor 2 (HER2), and CD133; the most used tumor-antigen for CAR-T cell development was EGFRVIII. The cell lines were studied in the included studies were U87MG, U251, DKMG, U373, and D270. U251 was the most studied cell line in the included preclinical studies. **Table 1** demonstrates the summarized data extracted from the included studies.

**Table 1:** The characteristics of included studies.

No.	First author, publication year	PD-1 disruption approach	Target of CAR-T	CAR-T generation	Cell line	Anti-PD-1 schedule in animal models	Animal model
1	Song et al., 2020 (9)	PD-1 antibody	EGFRVIII	Second-generation	U87	14 to 21 days after tumor inoculation (once the majority of tumors exhibited an area greater than 100 mm2)	6- to 8-week-old female immunodeficient NPI mice
2	Nakazawa et al., 2020 (11)	CRISPR/Cas9-mediated gene therapy	EGFRVIII	Third-generation	U-251MG and DKMG	Not applicable	Not applicable
3	Portnow et al., 2020 (8)	PD-1 antibody	HER2 and IL13Rα2	Not mentioned	Not applicable	Not applicable	Not applicable
4	Zhu et al., 2020 (18)	PD-1 siRNA-mediated gene therapy	EGFRVIII	Third-generation	U373	Not applicable	BALB/c nude mice
5	Shen et al., 2019 (6)	PD-1 antibody	HER2	Third-generation	U251 and U87	Not applicable	Not applicable
6	Choi et al., 2019 (10)	CRISPR/Cas9-mediated gene therapy	EGFRVIII	Second-generation	U87 and U251	Not applicable	Immune compromised NSG mice
7	Hu et al., 2019 (19)	The nucleofection of plasmid DNA for CRISPR/Cas9-mediated gene therapy	CD133	Third-generation	U251	Not applicable	6- to 8-week-old female NPG mice
8	Yin et al., 2018 (20)	PD-1 antibody	IL13Rα2	Second-generation	U87, U251, and D270	From day 6 after tumor implantation	6- to-8-week-old female NSG mice

### 3.3 Anti-PD-1 Can Substantially Increase the Survival of Glioma Animal Models on Second-Generation CAR-T Cells

**Figure 2:**
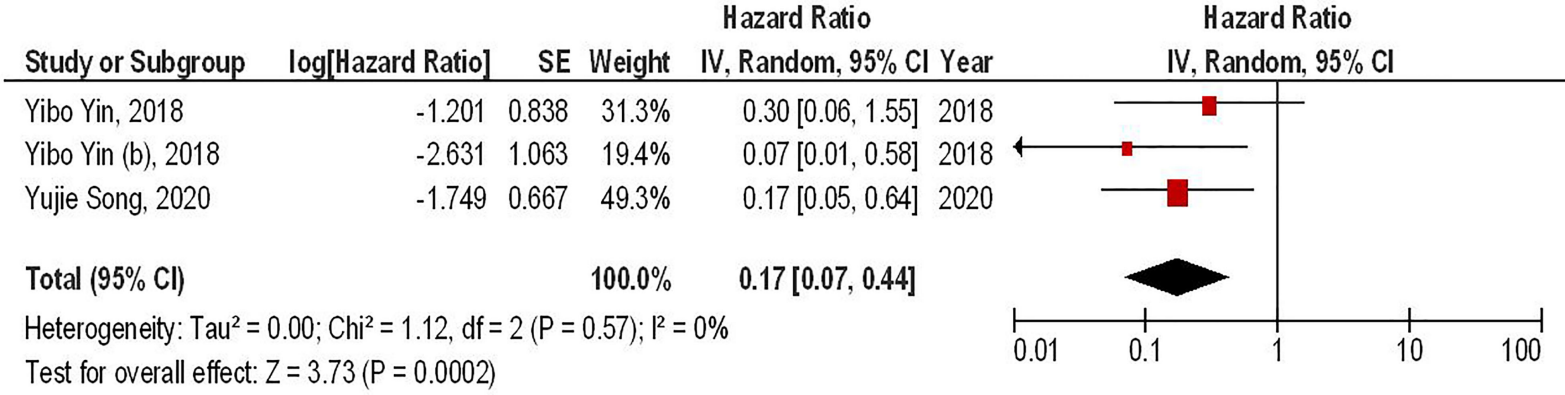
The forest plot of studies evaluating the effect of anti-PD-1 administration on the survival of animal models treated with second-generation CAR-T cells.

Our results have demonstrated that the combined therapy of anti-PD-1 with second-generation CAR-T cells can significantly enhance the survival of animal-bearing gliomas compared to the monotherapy with second-generation CAR-T cells (HR = 0.17, 95% CI: 0.07 - 0.44, P = 0.0002). Besides, there has been no significant heterogeneity between the included studies (I^2^ = 0%, P = 0.75) (**Figure 2**).

### 3.4 PD-1 Knockdown Can Remarkably Increase the Survival of Glioma Animal Models on CAR-T Cells

**Figure 3:**
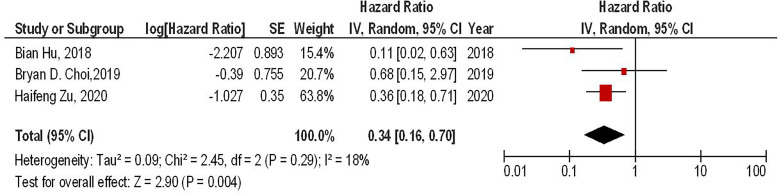
The forest plot of studies evaluating the effect of PD-1 knockdown on the survival of animal models treated with CAR-T cells.

Our results have shown that regardless of the CAR-T generations, the PD-1 gene-edited CAR-T cells can significantly improve the survival of glioma animal models compared to the conventional CAR-T cells (HR = 0.34, 95% CI: 0.16 - 0.70, P = 0.004). Also, no significant heterogeneity between the included studies has been found (I^2^ = 18%, P = 0.29) (**Figure 3**).

### 3.5 PD-1 Knockdown Can Considerably Increase the Survival of Glioma Animal Models on Third-Generation CAR-T Cells

**Figure 4:**
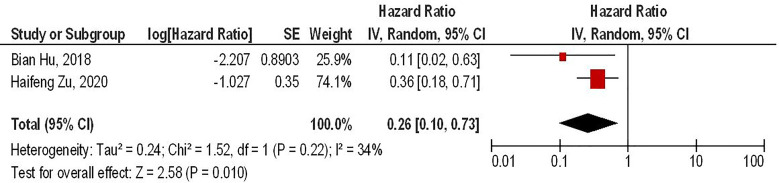
The forest plot of studies evaluating the effect of PD-1 knockdown on the survival of animal models treated with third-generation CAR-T cells.

Our results have shown that PD-1 gene-edited third-generation CAR-T cells can significantly improve the survival of glioma animal models compared to the conventional third-generation CAR-T cells (HR = 0.26, 95% CI: 0.10 - 0.73, P = 0.01). Besides, no significant heterogeneity between the included studies has been noted (I^2^ = 34%, P = 0.22) (**Figure 4**).

### 3.6 Evaluating Publication Bias

**Figure 5:**
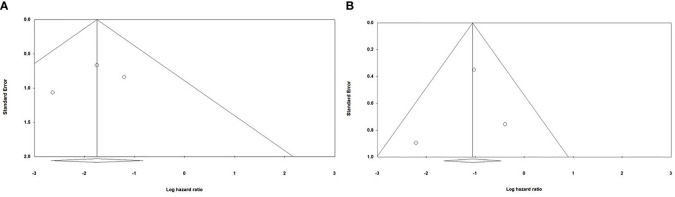
Evaluating potential publication bias among the included studies **(A)** Evaluating publication bias among the studies investigating the effect of anti-PD-1 administration on the survival of animal models treated with second-generation CAR-T cells; Begg and Mazumdar’ test one-tail P-value=0.30075 and two-tail P-value =0.60151; Egger’s test one-tail P-value=0.3272 two-tail P-value=0.65456 **(B)** Evaluating publication bias among the studies investigating the effect of PD-1 knockdown on the survival of animal models treated with CAR-T cells; Begg and Mazumdar’ test one-tail P-value=0.30075 and two-tail P-value =0.60151; Egger’s test one-tail P-value=0.40773 two-tail P-value=0.81545.

Begg and Mazumdar’s and Egger’s tests were performed to evaluate the asymmetry of funnel plots and potential publication bias. Our results have demonstrated that asymmetry is not present in the funnel plots, and there is no publication bias that can affect the obtained results (**Figure 5**).

### 3.7 Evaluating the Bias in the Included Studies

**Table 2** evaluates the potential bias in the included clinical study based on the criteria of the NIH quality assessment tool. Overall, no considerable bias has been noted. **Table 3** assesses the potential bias in the included *in vitro* studies. Based on our results, the risk of potential bias is considered low. **Table 4** evaluates the potential bias among the *in vivo* investigations. The primary bias domains have been randomly selecting the animal models and their housing. Besides, only one *in vivo* study has evaluated the side effects of treatments, i.e., lymphoma development and graft versus host disease, in the mice (19).

**Table 2:** Evaluating the potential risk of bias in the included clinical study.

Items	Yes	No	Other (CD, NR, NA)*
1. Was the study question or objective clearly stated?	*		
2. Were eligibility/selection criteria for the study population prespecified and clearly described?	*		
3. Were the participants in the study representative of those who would be eligible for the test/service/intervention in the general or clinical population of interest?	*		
4. Were all eligible participants that met the prespecified entry criteria enrolled?	*		
5. Was the sample size sufficiently large to provide confidence in the findings?		*	
6. Was the test/service/intervention clearly described and delivered consistently across the study population?	*		
7. Were the outcome measures prespecified, clearly defined, valid, reliable, and assessed consistently across all study participants?	*		
8. Were the people assessing the outcomes blinded to the participants’ exposures/interventions?			*
9. Was the loss to follow-up after baseline 20% or less? Were those lost to follow-up accounted for in the analysis?			*
10. Did the statistical methods examine changes in outcome measures from before to after the intervention? Were statistical tests done that provided p values for the pre-to-post changes?	*		
11. Were outcome measures of interest taken multiple times before the intervention and multiple times after the intervention (i.e., did they use an interrupted time-series design)?	*		
12. If the intervention was conducted at a group level (e.g., a whole hospital, a community, etc.), did the statistical analysis take into account the use of individual-level data to determine effects at the group level?	*		

*CD, cannot determine; NA, not applicable; NR, not reported.

**Table 3:** Evaluating the potential risk of bias in the included *in vitro* investigations.

No.	First author, publication year	1. Was the studied cancer cell lines reported?	2. Was the duration of exposure to the CAR-T cells to tumoral cells reported?	3. Was the concentration of the studied CAR-T cells reported?	4. Was a standard culture media used for the study?	5. Were reliable tools used to assess the outcome?	6. Were the experiments conducted more than once?	7. Were more than one independent experiment performed?	The overall risk of bias
1	Nakazawa et al., 2020 (11)	Yes	Yes	Yes	Yes	Yes	Yes	Yes	Low
2	Shen et al., 2019 (6)	Yes	Yes	Yes	Yes	Yes	Yes	Yes	Low

**Table 4:** Evaluating the potential risk of bias in the included *in vivo* investigations.

No.	First author and publication year	Sequence generation	Baseline characteristics	Allocation concealment	Random housing	Blinding (performance bias)	Random outcome assessment	Blinding (detection bias)	Incomplete outcome data	Selective outcome reporting	Other sources of bias
1	Song et al., 2020 (9)	***	***	***	**	**	***	**	***	**	Not noted
2	Zhu et al., 2020 (18)	***	***	***	**	**	***	**	***	**	Not noted
3	Choi et al., 2019 (10)	***	***	***	**	**	***	**	***	**	Not noted
4	Hu et al., 2019 (19)	***	***	***	**	**	***	**	***	***	Not noted
5	Yin et al., 2018 (20)	***	***	***	**	**	***	**	***	**	Not noted

***Not bias might be noted; **A slight bias might be noted; *Obvious bias might be noted.

## 4 Discussion

The dismal prognosis of high-grade glioma patients with the current therapy requires developing new strategies to target cancer cells. Although CAR-T cells have demonstrated clinical benefit for patients with B-cell malignancies, this technology has not been that successful for patients with high-grade gliomas. The immunosuppressive tumor microenvironment and tumor heterogeneity are among the culprits of this failure. Because CAR-T administration has been associated with the upregulated expression of inhibitory immune checkpoint molecules in the CAR-T and tumoral cells, targeting inhibitory immune checkpoints, such as the programmed death-ligand 1 (PD-L1)/PD-1 axis, has shown promising results (9). In this regard, this systematic review and meta-analysis aimed to investigate the current approaches to target PD-1 expression in CAR-T cells, i.e., monoclonal antibody administration for targeting PD-1 and PD-1 gene-editing of CAR-T cells in high-grade glioma.

### 4.1 PD-1 Blockade and PD-1 Gene-Editing in CAR-T Cells for High-Grade Gliomas: What Does the Currently Available Evidence Say?

Our meta-analysis has indicated that co-administrating monoclonal antibodies for targeting PD-1 with second-generation CAR-T cells can significantly improve the survival of glioma-animal models compared to monotherapy with second-generation CAR-T cells (HR = 0.17, 95% CI: 0.07 - 0.44, P = 0.0002). It has been reported that administrating PD-1 inhibitors can remarkably increase the infiltration of immune cells into the tumor microenvironment and upregulate the expression of interleukin-2 (IL-2) and interferon-gamma (IFN-γ) (6, 9). Consistent with these, anti-PD-1 administration has been associated with a considerable decrease in the tumor size in mice bearing glioma (20). Of interest, a recent clinical trial has indicated that intravenous pembrolizumab, an anti-PD-1 monoclonal antibody, can result in a steady-state concentration of pembrolizumab in the cerebrospinal fluid (CSF) and suppress the PD-1 expression in CAR-T cells (6). Moreover, intravenous pembrolizumab can inhibit PD-1 expression in non-CAR T-cells, indicating its inhibitory role on other tumor-infiltrative immune cells, e.g., regulatory T cells (8). Consistent with the clinical study results, pembrolizumab can increase the persistency and anti-tumoral activity of CAR-T cells in patients with relapsed B-cell acute lymphoblastic leukemia (21). Since PD-1 is expressed by CAR-T cells and other immune cells in the tumor microenvironment, its blockade might be a promising strategy to increase the efficacy of CAR-T cells.

Our results have also demonstrated that regardless of the CAR-T generations, PD-1 gene-edited CAR-T cells can significantly improve the survival of glioma-animal models compared to the conventional CAR-T cells (HR = 0.34, 95% CI: 0.16 - 0.70, P = 0.004). Besides, PD-1 gene-editing of third-generation CAR-T cells can significantly improve the survival of glioma-animal models compared to the conventional third-generation CAR-T cells (HR = 0.26, 95% CI: 0.10 - 0.73, P = 0.01). Based on the limited currently available data, PD-1 gene-edited CAR-T cells do not lead to lymphoma development and graft versus host disease in mice bearing glioblastoma (17). Nevertheless, further investigations are needed to evaluate the safety of these approaches. Furthermore, PD-1 deletion has been associated with increased central memory T-cell-like properties, leading to elevated proliferation, increased persistence, and self-renewal features in glioblastoma (10). PD-1 deletion has also upregulated the expression of pro-inflammatory cytokines, i.e., IL-2, IFN-γ, and tumor necrosis factor-alpha (TNF-α), which are associated with increased anti-tumoral immune responses against glioblastoma (10, 18). Zhu et al. have shown that the increased anti-tumoral immune responses of PD-1 gene-edited CAR-T cells are more pronounced against glioblastoma that overexpress PD-L1 (18). Thus, tumoral PD-L1 expression might be a prognostic factor for this approach.

### 4.2 The Limitations of PD-1 Blockade and PD-1 Gene Editing

#### 4.2.1 Anti-PD-1 in Treating Gliomas; One Piece of a Big Puzzle?

Although the currently available evidence has suggested that suppressing PD-1 can substantially increase the efficacy of CAR-T cells, the tumor-microenvironment is usually more complicated than its fate can be attributable to a single inhibitory immune checkpoint molecule. Indeed, other inhibitory immune checkpoints, e.g., cytotoxic T-lymphocyte-associated protein 4 (CTLA-4), T cell immunoglobulin domain and mucin domain-3 (TIM-3), V-domain Ig suppressor of T cell activation (VISTA), lymphocyte activation gene 3 (LAG-3), PD-L1, and T cell immunoreceptor with Ig and ITIM domains (TIGIT), can also promote an immunosuppressive tumor microenvironment (4, 22). This has been reflected in the multiple clinical trials investigating the efficacy of immune checkpoint inhibitors in glioma patients. Pembrolizumab administration has resulted in no clinical/histologic improvements in patients with brain tumors (23). Nayak et al. have shown that the objective response rate of patients with recurrent glioblastoma to pembrolizumab is 0% (24). A recent phase 3 randomized clinical trial has also demonstrated that the response rate of glioblastoma patients to nivolumab, another anti-PD-1 monoclonal antibody, is poor, and the objective response rate of affected patients to this anti-PD-1 agent is 7.8% (25). Consistent with these, Omuro et al. have reported that the complete response rate of patients to nivolumab is 0%. Not only adding ipilimumab, an anti-CTLA-4 antibody, to the nivolumab regimen has not improved the complete response rate of patients with recurrent glioblastomas, but also the co-administration of ipilimumab and nivolumab has been associated with increased occurrence of treatment-induced adverse events (26). Indeed, administrating multiple inhibitory immune checkpoint inhibitors has been associated with an increased risk of autoimmunity development; because it paves the way for stimulating auto-reactive T cells. Matull et al. have reported that combined CTLA-4 and PD-1 inhibition can severely damage multiple organs following a single dosage of ipilimumab and nivolumab (27). Simonelli et al. have shown that nivolumab, as a PD-1 inhibitor, can severely damage the liver in a glioblastoma patient (28). Thummalapalli et al. have reported that suppressing PD-1 and indoleamine-pyrrole 2,3-dioxygenase (IDO) can lead to hemophagocytic lymphohistiocytosis, acute liver injury, cytopenia, and altered mental status in a patient with recurrent glioblastoma (29). A recent clinical trial has shown that 18.1% of patients with recurrent glioblastoma have manifested grade 3/4 treatment-related adverse events following nivolumab administration (25). In line with these, a recent systematic review has indicated that CTLA-4 inhibitors can promote immune-related adverse events and lead to organ-specific damage (30). Therefore, the safety issues of the current method of immune checkpoint inhibitors administration might be a daunting challenge.

As discussed above, these unfavorable results to targeting one inhibitory immune checkpoint molecule might indicate that a network of the inhibitory immune checkpoint can regulate anti-tumoral immune responses, and targeting one axis can lead to the compensation of this network *via* other inhibitory immune checkpoint molecules. Indeed, the reason for the relatively favorable response rate of animal models or affected patients to immune checkpoint inhibitors can be stemmed from the fact that a specific inhibitory immune checkpoint molecule plays a predominant role in that network. Yin et al. have reported that anti-CTLA-4 administration has been associated with prolonged survival of glioma models treated with Hu08BBz compared to the administration of anti-TIM-3. However, the anti-PD-1 administration has been more effective in improving the survival of glioma models treated with 2173BBz, a second-generation CAR-T cell agonist EGFRVIII, compared to anti-CTLA-4 administration (20). In line with these, the response rates of affected patients to a specific immune checkpoint inhibitor vary substantially, and the overall response rate of glioblastoma patients is not favorable. A phase I clinical trial has demonstrated that the overall response rate of glioblastoma patients with positive tumoral PD-L1 to pembrolizumab has been 8% (31).

#### 4.2.2 The Shortcomings of PD-1 Gene-Edited CAR-T Cells for Treating Gliomas

PD-1 gene-editing also harbors shortcomings. Single inhibitory immune checkpoint gene-editing of CAR-T cells cannot disrupt the inhibitory immune checkpoint axes between other cells residing in the tumor microenvironment. Besides, PD-1 disruption has slightly demonstrated off-target effects *via* targeting the T cell-related growth factor genes; thus, the proliferation of PD-1 gene-edited CAR-T cells can be slightly decreased compared to non-edited immune cells (11). In contrast, Song et al. have indicated that PD-1-targeting monoclonal antibody administration is not associated with decreased proliferation of CAR-T cells in glioblastoma (9). Therefore, special percussions are needed in developing gene-edited CAR-T cells *via* CRISPR/Cas9 technology to avoid the off-target effect. Also, siRNA-mediated PD-1 knockdown can be time-dependent; thus, PD-1-siRNA degradation can promote PD-1 upregulation on CAR-T cells. Therefore, further research is needed to address the gene-editing of PD-1 at the post-transcriptional level and deleting the PD-1 gene itself.

### 4.3 Glioblastoma Treatment in the Era of Single-Cell Sequencing and Fourth-Generation CAR-T Cells

#### 4.3.1 How Can Single-Cell Sequencing Further Our Knowledge of the Very Dynamic Nature of the Tumor Microenvironment?

Single-cell sequencing technologies have revolutionized our knowledge of the cells that reside in the tumor microenvironment. Recently, Fu et al. have shown a substantial increase in the level of tumor-infiltrating TIM-3^+^CD8^+^ and PD-1^+^CD8^+^ T-cells in anaplastic astrocytoma tissues compared to corresponding cells in the peripheral blood mononuclear cells (PBMCs) from affected patients. The same trend has been true for tumor-infiltrative CD4^+^ T-cells. These phenotypically exhausted T-cells, along with the increased level of Treg infiltration, can participate in the immunosuppressive tumor microenvironment development (32). Consistent with this, Davidson et al. have demonstrated that PD-1, LAG-3, and TIM-3 are substantially upregulated in tumor-infiltrating CD3^+^ T-cells compared to corresponding cells of the PBMCs of glioma and normal individuals. Nevertheless, the tumor-infiltrating lymphocytes upregulate the expression of the genes involved in T-cell activation, i.e., CD38 and HLA-DR, and the genes pertained to T-memory phenotype, i.e., CD45RA, CD27, and CD127, compared to PBMCs of glioma patients (33). Therefore immune cells express both stimulatory and inhibitory molecules, and the traditional categorizing of immune cells based on one inhibitory immune checkpoint might not reveal the role of those immune cells. Besides, inhibitory immune checkpoint molecules, e.g., PD-1, can be transiently expressed following immune cells activation (34). In line with this, Clarke et al. have demonstrated that despite TIM-3 and PD-1 expression, tissue-resistant memory T-cells have demonstrated remarkable proliferation and upregulation of pro-inflammatory genes in lung cancer (35). In breast cancer patients, Bassez  et al. have indicated that T-cells with PD-1, HAVCR2, LAG-3, and CD39 phenotype can substantially expand despite the expression of exhaustion-related markers. This phenomenon might be attributable to the fact that these cells also express the cytotoxic-related markers, antigen-presenting markers, and immune cell homing signals as well (36). In melanoma, Deng et al. have shown that cytotoxic subpopulation of CD8^+^ T-cells, associated with improved prognosis, also demonstrate relatively increased expression of inhibitory immune checkpoint molecules, i.e., CTLA4, LAG3, PD-1, HAVCR2, and TIGIT (37). Thus, the expression of several inhibitory immune checkpoints does not always reflect attenuated anti-tumoral immune response, and a network of genes is involved in the fate of anti-tumoral immune responses.

The data obtained from the single-cell sequencing can help us decipher the unfavorable and variable response rate of affected patients to immune checkpoint inhibitors as well. Durante et al. have demonstrated that cytotoxic T lymphocytes (CTLs) do not overexpress PD-1/CTLA-4 molecules rather LAG-3 and provide evidence for the low response rate of uveal melanoma to the current version of immune checkpoint inhibitors (38). Darmanis et al. have demonstrated that only a minority of glioblastoma patients express the ligands for PD-1 and CTLA-4 on tumoral cells, which might be the reason for the overall unfavorable response rate of glioblastoma patients to anti-PD-1 and anti-CTLA-4 agents (39). Consistent with these, Yin et al. have shown that the administration of anti-CTLA-4 with Hu08BBz, a second-generation CAR-T cell against IL-13Rα2, is more effective in improving the survival of mice bearing glioma compared to the administration of anti-PD-1 (20). Indeed the different inhibitory immune checkpoint profiles of the tumor microenvironment, which can be different from case to case, might be the underlying reason for these disparities. A recent clinical trial has shown that the increased level of CD68^+^ macrophages, which have strong associations with VISTA and B7-H3 expression, can be the underlying reason for the low response rate of glioblastoma patients to pembrolizumab (40). Therefore, the dynamic intercellular cross-talk in the tumor microenvironment can be implicated in the low response rate of glioma patients to immune checkpoint inhibitors.

Moreover, single-cell sequencing can identify specific tumor biomarkers for determining the response rate of affected patients to immune checkpoint inhibitors. It has been reported that the TCF7 expression in CTLs can be a valuable prognostic factor for determining the response rates of melanoma patients to anti-PD-1 therapy (41). Furthermore, single-cell sequencing can help us identify novel inhibitory immune checkpoints. Li et al. have reported that sialic acid-binding Ig-like lectin-5, sialic acid-binding Ig-like lectin-7, sialic acid-binding Ig-like lectin-9, and sialic acid-binding Ig-like lectin-16 can be considered novel inhibitory immune checkpoint molecules that are functionally similar to TIM-3 and PD-L1. Besides, their combined inhibition might improve the prognosis of glioma patients (42). Collectively, single-cell sequencing can further our understanding of the tumor microenvironment.

#### 4.3.2 Tumoral Antigen for CAR-T Cells and Single-Cell Sequencing

Identifying tumor-specific antigens for developing CAR-T cells might be one of the daunting challenges because of temporal, intra-, and inter-tumoral heterogeneity in the tumor bulk. This justifies the identification of multiple (neo-) antigens for each affected patient. Nejo et al. have classified tumoral antigen into four groups, i.e., virus-derived antigen, patient-specific neoantigen, shared neoantigen, and non-mutant shared antigen. EGFRVIII is an example of the shared neoantigens for glioma, and IL13Rα2 and HER2 are examples of the non-mutant shared antigens that our study has shown that CAR-T cells have been designed against them (43). Since discussing all aspects of these categories is out of the scope of the current study, we discuss the advantages and disadvantages of non-mutant shared antigens, shared neoantigens, and patient-specific neoantigen and highlight how single-cell sequencing data can improve the efficacy of CAR-T therapies.

##### 4.3.2.1 Non-Mutant Shared Antigens: Time to Re-Think About Their Safety?

One of the advantages of this approach is that these antigens can be considered as “off-shelf.” However, their relatively low specificity is the main disadvantage of this approach. The expression levels of non-mutant shared antigens are substantially higher in tumoral cells compared to normal cells. Besides, non-mutant shared antigens can be overexpressed in other malignancies as well as glioblastoma. For instance, HER2 can be overexpressed in pancreatic cancer, lung adenocarcinoma, and breast cancer (44–46). However, due to the vast temporal, intra-, and inter-tumoral heterogeneity in tumor bulk and application of immunohistochemistry (IHC) rather than investigating tumor bulk at single-cell levels, it is difficult to prescribe one non-mutant shared antigen for patients with a specific malignancy. Besides, non-mutant shared antigens can be expressed in normal cells at physiological levels, and the related CAR-T cells can severely damage normal tissues. For instance, Morgan et al. have reported a metastatic colorectal cancer patient treated with anti-HER2-CAR-T cells and developed cytokine release syndrome and respiratory distress after transfusion of CAR-T cells. This phenomenon might be stemmed from the fact that HER2 can be expressed in lung epithelial as well (47). Also, it has been shown that IL-13Rα2-targeting CAR-T cells can develop anti-tumoral immune responses against aortic and pulmonary artery smooth in glioma animal models (20). Therefore, developing CAR-T cells against non-mutant shared antigens can increase the risk of adverse events in affected patients.

##### 4.3.2.2 Shared Neoantigens: Does Tumor Evolution Lead to Its Evasion?

Shared neoantigens can also be considered “off-shelf,” and their high specificity is another advantage. However, tumor cells mutate, which leads to their evasion from the cytotoxic machinery of highly specific CAR-T cells. A recent clinical trial has shown that the expression level of EGFRVIII is substantially decreased following anti-EGFRVIII CAR-T cells infusion; however, the anti-EGFRVIII CAR-T cells have not entirely eradicated glioma cells (7). Zhu et al. have also demonstrated that although PD-1 gene-edited anti-EGFRVIII CAR-T cells can decrease glioma growth in affected mice, these CAR-T cells also can not entirely eradicate tumoral cells (18). Krenciute et al. have shown that developed genetically engineered CAR-T cells to express IL-15. Although these genetically modified CAR-T cells have demonstrated increased persistence and anti-tumoral effects in glioma-animal models, their efficacy has been limited over time. Because treating glioma cells with CAR-T cells that only target one tumoral (neo-) antigen can lead to (neo-) antigen loss in tumoral cells (48). Consistent with these, Bielamowicz et al. have reported that treating glioma with CAR-T cells with three different molecular targets can exhibit higher cytotoxicity. Besides, animal models treated with CAR-T cells with three different molecular targets have experienced more prolonged survival than those treated with one molecular target (49). Collectively, tumoral cells exhibit vast heterogeneity, and administrating multiple CAR-T cells that target multiple neoantigens can yield optimal results.

##### 4.3.2.3 Patient-Specific Neoantigens and Single-Cell Sequencing in the Era of Personalized Medicine

Patient-specific neoantigens are the results of the genetic alteration of each patient. The main advantage of these neoantigens is that the immune system does not exhibit considerable tolerance against them, and normal cells do not physiologically express them. Nevertheless, identifying these neoantigens might be a daunting challenge. Besides, the relatively low mutation rate of glioblastoma distinguishes it from other cancers, leading to low tumoral neo-antigen development (50). In this regard, single-cell sequencing of tumor bulk can help identify (potential) patient-specific neoantigens. Single-cell sequencing technologies can provide valuable insights into the expression profile of tumoral cells and categorize tumoral cells based on their neoantigens (15, 51). Therefore, this categorization can allow us to develop personalized CAR-T cells with different molecular targets for each patient. In this approach, the vast intra- and inter-heterogeneity of glioma cells can be addressed, and the subsequent tumor recurrence can be prevented.

Nevertheless, single-cell sequencing-guided CAR-T cell generation harbors some limitations as well. One of the main disadvantages of this approach is that it is not “off-shelf,” and its rapid availability requires further consideration and implantation of high-tech centers. Besides, the conventional single-cell sequencing method is based on RNA sequencing; however, the mRNA expression level is not always well-correlated with its protein expression level. In this regard, applying RNA expression and protein sequencing (REAP-seq) and antibody sequencing can address this issue. Also, despite its promising future in eradicating glioma cells, the proposed strategy might be expensive, and assessing its cost-effectiveness requires further investigations (52). Lastly, the excessive immunosuppressive tumor microenvironment of glioma can substantially suppress the stimulation of CAR-T cells-mediated anti-tumoral immune responses even though the CAR-T cells are specifically designed for patient-derived antigens. For this issue, we propose single-cell sequencing-guided fourth-generation CAR-T cell development (see below).

#### 4.3.3 The Combination of Single-Cell Sequencing and Fourth-Generation of CAR-T Cells: A New Perspective for Treating Glioblastoma?

Compared to systemic administration of multiple immune checkpoint inhibitors to reverse the immunosuppressive tumor microenvironment, the application of fourth-generation CAR-T cells can be promising in terms of decreasing the risk of immune-related adverse events development. In this approach, the stimulation of CAR-T cells can lead to the expression and release of desired factors in the microenvironment. This generation has shown encouraging results in expressing intended factors following the stimulation. Lanitis et al. have demonstrated that fourth-generation CAR-T cells can transform the immunosuppressive tumor microenvironment into a pro-inflammatory one, confer enhanced anti-tumoral immune responses, upregulation of B-cell lymphoma 2 (Bcl-2) in CAR-T cells, and activate natural killer cells *via* IL-15 expression (53). Daun et al. have shown that administration of fourth-generation CAR-T cells expressing IL-7 and CCL19 can remarkably increase the migration and cytotoxicity of CAR-T cells against multiple myeloma and substantially reduce urine protein-light levels in affected patients (54). Mei et al. have developed fourth-generation MUC-1-targeting CAR-T cells that release IL-22. They have shown that IL-22 release can considerably increase MUC-1 expression in head and neck squamous cell carcinoma cells; however, this effect mostly halted after 72 hours. Nevertheless, they have shown that these fourth-generation CAR-T cells can substantially decrease tumor volume and increase the infiltration of CD3^+^ T-cells in animal models (55). A recent clinical trial has demonstrated that administration of CD19-targeting fourth-generation CAR-T cells to relapsed/refractory B cell non-Hodgkin lymphoma patients with the life-expediency of fewer than two months can lead to the median overall survival of 23.8 months. The overall response rate of the affected patients to these CAR-T cells has been 67%; however, the incidence of cytokine release syndrome development has been 14% (56).

**Figure 6:**
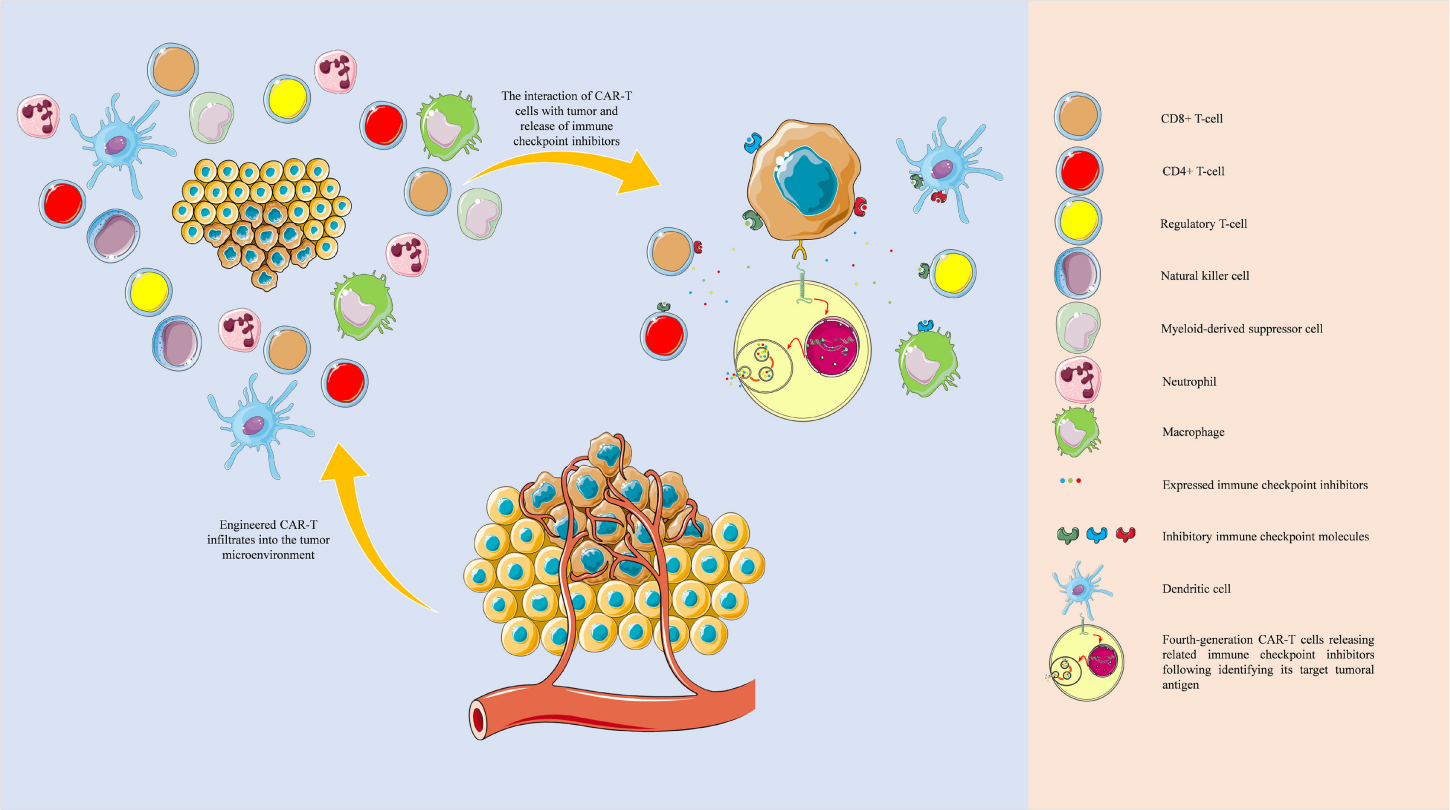
Tumor microenvironment and single-cell sequencing-guided fourth-generation CAR-T cells. The development of fourth-generation CAR-T cells based on the single-cell sequencing-identified patient-derived neoantigens and the single-cell sequencing-guided inhibitory immune checkpoint molecules profiling can potentially eradicate tumoral sub-populations and effectively attenuate inhibitory immune checkpoint network present in the tumor microenvironment. The objects of this figure were obtained from https://smart.servier.com/.

Overall, one of the main advantages of fourth-generation CAR-T cells over others is their stimulatory effect on the “bystander” cells in the tumor microenvironment, which liberates them from exhaustion. The same concept can be applied for expressing inhibitory immune checkpoint inhibitors (**Figure 6**). Zhou et al. have engineered a fourth-generation CAR-T, EGFR BB-z/E30-CAR-T, that can express and release PD-1-targeting antibodies following its stimulation. This fourth-generation CAR-T cell has demonstrated higher anti-tumoral immune responses and tumor infiltration rates than its corresponding second-generation one (57). Krenciute et al. have shown that IL13Rα2-targeting CAR-T cells, which express IL-15, can substantially demonstrate higher anti-tumoral immune responses (48). Nevertheless, genetically modified CAR-T cells are also prone to failure; because treating glioma cells with CAR-T cells that only target one tumoral antigen can result in antigen loss in tumoral cells. Therefore, there is a need to develop multiple types of CAR-T cells that their molecular targets are specifically expressed in all sub-populations of tumoral cells, i.e., patient-specific neoantigens. With the obtained data from the single-cell sequencing of cells in the tumor microenvironment, we can design multiple types of CAR-T cells that can cover tumoral neoantigens expressed in various tumor cell sub-populations and express the related immune checkpoint inhibitors following stimulation (**Figure 6**). In this approach, the pertained immune checkpoint inhibitors are released in the tumor microenvironment, which does not increase the risk of autoimmunity development in other organs. For this purpose, an atlas of neoantigens and inhibitory immune checkpoint molecules of the tumor microenvironment might be needed to link the certain phenotype of the patient’s tumor with the personalized CAR-T cells. Therefore further studies are needed to build such an atlas, and the application of machine learning and artificial intelligence can facilitate this process. Besides, it remains to be determined how effective the single-cell sequencing-guided fourth-generation CAR-T cells approach can be; because it has been indicated that glioblastoma can induce systemic immunosuppression and T cell dysfunction (58–60). Therefore, further studies are needed before the translation of this approach into clinical practice.

### 4.4 The Current Trend of Clinical Trials of CAR-T Cells for Treating Patients With High-Grade Glioma

Based on our discussion, the combination of fourth-generation CAR-T cells with the data of single-cell sequencing of tumoral cells and cells residing in the tumor microenvironment can substantially improve anti-tumoral immune responses. Regarding the application of CAR-T cells in treating high-grade gliomas, the current trend in the clinical trials is summarized in **Table 5**. Although the combination of radiation/cytotoxic agents can considerably promote the immunogenicity of glioma cells *via* activating damage-associated molecular pattern (DAMP) signalings and promoting local inflammation, chemo-/radioresistance might attenuate the efficacy of this strategy. Therefore, further investigations for nurturing this combination therapy might be needed (61, 62). NCT04003649 clinical trial is the phase I clinical trial that investigates the combination of IL13Rα2-CAR T cells with nivolumab and ipilimumab in patients with recurrent/refectory glioblastoma. Based on the current evidence discussed in this study, potential immune resistance, tumor relapse, and low-response rates might be challenging.

**Table 5:** The current trend in treating the high-grade glioma patients with CAR-T-based therapy.

No.	Intervention	Cancer type	Clinical trial phase	(estimated) study start date	The status	Clinicaltrials.gov Identifier
1	B7-H3 CAR-T + Temozolomide	Recurrent/refectory glioblastoma	Phase I	1-Jun-20	Recruiting	NCT04385173
2	NKG2D CAR-T	Recurrent glioblastoma	Not applicable	1-Sep-21	Not yet recruiting	NCT04717999
3	B7-H3 CAR-T + Temozolomide	Recurrent/refectory glioblastoma	Phase I/II	1-May-22	Recruiting	NCT04077866
4	GD2 CAR-T + Fludarabine + Cyclophosphamide	Glioma of spinal cord/glioma of brainstem	Phase I	4-Jun-20	Recruiting	NCT04196413
5	CD147-CAR-T	Recurrent CD147 positive glioblastoma	Early phase I	30-May-19	Recruiting	NCT04045847
6	IL13Rα2-CAR-T + Nivolumab + Ipilimumab	Recurrent/refectory glioblastoma	Phase I	26-Sep-19	Recruiting	NCT04003649
7	CAR-T + Radiation + TCR-T + GM-CSF	High-grade glioma	Phase I	1-Apr-18	Recruiting	NCT03392545
8	CAR-T	Recurrent malignant glioma	Phase I	2-Mar-18	Recruiting	NCT03423992
9	IL13Rα2-CAR-T Cell	Leptomeningeal metastases of glioblastoma	Phase I	15-Feb-21	Recruiting	NCT04661384
10	B7-H3 CAR-T	Diffuse glioma	Phase I	11-Dec-19	Recruiting	NCT04185038
11	Fludarabine + Cyclophosphamide + C7R-GD2.CAR-T	High-grade glioma	Phase I	3-Feb-20	Recruiting	NCT04099797

The current study has some strengths. First, we have used a systematic and unbiased approach to identify and summarize the currently available evidence on the significance of co-administration of anti-PD-1 with CAR-T cells and PD-1 gene-editing of CAR-T cells for glioma therapy. Second, we included both preclinical and clinical studies, carefully evaluated their potential bias based on pertained checklists, and attempted to sort out the inconsistencies between these two. Third, we objectively evaluated the efficacy of these two approaches in improving the survival of animal models *via* applying multiple tests for assessing between-study heterogeneity and publication bias. Fourth, there has not been remarkable between-studies heterogeneity that poses questions about the significance of the interventions. Fifth, we proposed a new strategy to ameliorate the response rate of CAR-T cells based on the detailed discussion on the recent preclinical and clinical findings regarding tumor microenvironment interactions and tumor antigens. However, the current study also suffers from several limitations. First, we only included papers published in English. Second, the protocol of the current study was not publicly available.

## 5 Conclusion

The co-administration of anti-PD-1 with CAR-T cells and PD-1 gene-editing of CAR-T cells can substantially prolong the survival of glioma-animal models, and anti-PD-1 can effectively accumulate in the CSF of patients with high-grade gliomas. However, clinical trials have failed to report favorable response rates of anti-PD-1 for glioblastoma patients, which might be due to the regulated inhibitory immune checkpoint network in the tumor microenvironment. Indeed, the fate of the tumor microenvironment is usually more complex than its direction can be determined by a single inhibitory immune checkpoint molecule. Currently available limited evidence has demonstrated that the gene-edited CAR-T cells might not be associated with severe side effects in animal models. To further increase the response rates of immune checkpoint inhibitors/CAR-T therapy, the combination of data obtained from single-cell sequencing of cells residing in the tumor microenvironment with fourth-generation CAR-T cells is suggested. The data from single-cell sequencing of tumoral cells can provide valuable insights into the patient-derived neoantigens that are specifically expressed in tumoral cells and cover subpopulations of tumoral cells. Also, the data from single-cell sequencing of cells residing in the tumor microenvironment can demonstrate the expression profile of inhibitory immune checkpoint molecules and their intensity in the tumor microenvironment, which can be used for engineering fourth-generation CAR-T cells to express the related immune checkpoint inhibitors following their stimulation. The proposed approach increases the chance of glioblastoma cells eradication. Also, because the immune checkpoint inhibitors are released in the tumor microenvironment, the risk of immune-related adverse events, seen following systemic administration of heavy dosage of multiple immune checkpoint inhibitors, might be decreased. Collectively, the combination of fourth-generation CAR-T cells with the data from single-cell sequencing technologies can open a new chapter in treating high-grade gliomas in the era of personalized medicine.

## Data Availability Statement

The original contributions presented in the study are included in the article/supplementary material. Further inquiries can be directed to the corresponding authors.

## Author Contributions

All authors have substantially contributed to the manuscript. MA: developing the research question, conducting the systematic search, conducting data extraction, performing the meta-analysis, conceptualization, and writing the manuscript. FB: helping with analysis. VK and NH: extracting data. AD, RF, and OB: providing the figures and tables. RB: reviewing the manuscript. BB and NS: reviewing the manuscript and supervising the project. All authors contributed to the article and approved the submitted version.

## Conflict of Interest

The authors declare that the research was conducted in the absence of any commercial or financial relationships that could be construed as a potential conflict of interest.

## Publisher’s Note

All claims expressed in this article are solely those of the authors and do not necessarily represent those of their affiliated organizations, or those of the publisher, the editors and the reviewers. Any product that may be evaluated in this article, or claim that may be made by its manufacturer, is not guaranteed or endorsed by the publisher.
